# Hydroxyethyl cellulose matrix applied to serial crystallography

**DOI:** 10.1038/s41598-017-00761-0

**Published:** 2017-04-06

**Authors:** Michihiro Sugahara, Takanori Nakane, Tetsuya Masuda, Mamoru Suzuki, Shigeyuki Inoue, Changyong Song, Rie Tanaka, Toru Nakatsu, Eiichi Mizohata, Fumiaki Yumoto, Kensuke Tono, Yasumasa Joti, Takashi Kameshima, Takaki Hatsui, Makina Yabashi, Osamu Nureki, Keiji Numata, Eriko Nango, So Iwata

**Affiliations:** 1 RIKEN SPring-8 Center, 1-1-1 Kouto, Sayo-cho, Sayo-gun, Hyogo 679-5148 Japan; 2 Department of Biological Sciences, Graduate School of Science, The University of Tokyo, 7-3-1 Hongo, Bunkyo-ku, Tokyo, 113-0033 Japan; 3 Division of Food Science and Biotechnology, Graduate School of Agriculture, Kyoto University, Gokasho, Uji, Kyoto 611-0011 Japan; 4 Institute for Protein Research, Osaka University, 3-2 Yamadaoka, Suita, Osaka 565-0871 Japan; 5 Department of Cell Biology and Anatomy, Graduate School of Medicine, The University of Tokyo, 7-3-1 Hongo, Bunkyo-ku, Tokyo, 113-0033 Japan; 6 Department of Physics, POSTECH, Pohang, 37673 Republic of Korea; 7 Department of Structural Biology, Graduate School of Pharmaceutical Sciences, Kyoto University, 46-29 Yoshida Shimoadachi-cho, Sakyo-ku, Kyoto 606-8501 Japan; 8 Department of Applied Chemistry, Graduate School of Engineering, Osaka University, 2-1 Yamadaoka, Suita, Osaka 565-0871 Japan; 9 Structural Biology Research Center, KEK High Energy Accelerator Research Organization, Tsukuba, Ibaraki 305-0801 Japan; 10 Japan Synchrotron Radiation Research Institute, 1-1-1 Kouto, Sayo-cho, Sayo-gun, Hyogo 679-5198 Japan; 11 Enzyme Research Team, Biomass Engineering Research Division, RIKEN Center for Sustainable Resource Science, Hirosawa, Wako-shi, Saitama 351-0198 Japan; 12 Department of Cell Biology, Graduate School of Medicine, Kyoto University, Yoshidakonoe-cho, Sakyo-ku, Kyoto 606-8501 Japan

## Abstract

Serial femtosecond crystallography (SFX) allows structures of proteins to be determined at room temperature with minimal radiation damage. A highly viscous matrix acts as a crystal carrier for serial sample loading at a low flow rate that enables the determination of the structure, while requiring consumption of less than 1 mg of the sample. However, a reliable and versatile carrier matrix for a wide variety of protein samples is still elusive. Here we introduce a hydroxyethyl cellulose-matrix carrier, to determine the structure of three proteins. The *de novo* structure determination of proteinase K from single-wavelength anomalous diffraction (SAD) by utilizing the anomalous signal of the praseodymium atom was demonstrated using 3,000 diffraction images.

## Introduction

Serial femtosecond crystallography (SFX) using ultrashort pulses from X-ray free-electron lasers (XFELs) can overcome typical radiation damage to protein crystals via the “diffraction-before-destruction” approach^1–7^. This has been used to obtain crystal structures of interesting proteins at room temperature^8–18^. Liquid jet injection of small protein crystals with continuous flow at relatively high speed (~10 m sec^−1^) is frequently exploited for serial sample loading^19^, consuming 10~100 mg of the sample. To reduce sample consumption, micro-extrusion techniques of specimens using viscous media such as a lipidic cubic phase (LCP)^20^, grease^21^, Vaseline (petroleum jelly)^22^ and agarose^23^ have been developed. These media can maintain a stable stream at a lower flow rate of 0.02~0.5 μl min^-1^ allowing sample consumption of less than ~1 mg. More recently, synchrotron-based serial crystallography has also been developed^22, 24, 25^, demonstrating that the sample loading technique with a viscous media becomes even more important in serial crystallography. This method with viscous media is technically simple, but some media produce stronger X-ray scattering that increase background noise. For data collection from small crystals (~1 μm), at atomic resolution, and *de novo* phasing with weak anomalous signals, a crystal carrier with low background scattering is essential to improve the signal-to-noise ratio^23^. To reduce background scattering from the carrier media, we introduced a hyaluronic acid matrix in SFX^26^. At the SPring-8 Angstrom Compact Free Electron Laser (SACLA)^27^, we operate an injector system under a helium atmosphere at 1 atm during micro-extrusion of the matrices^28^. However, hyaluronic acid matrix is strongly adhesive, resulting in frequent clogging of the sample-vacuum nozzle which acts as a sample catcher^22^ in our injector system. In addition, the general adaptability of hydrogel matrices to *de novo* phasing with heavy atoms is still unclear.

Here we introduce hydroxyethyl cellulose (cellulose matrix) for serial sample loading. We demonstrate the cellulose matrix as a protein carrier for SFX using small and large sized crystals (1 × 1 × 1 to 20 × 20 × 30 μm). In addition, we demonstrate the successful *de novo* phasing in SFX by applying praseodymium (Pr)-SAD, single-isomorphous replacement (SIR) and SIR with anomalous scattering (SIRAS) phasing to determine the structure of proteinase K. Furthermore, to reduce background scattering, a novel grease matrix, Super Lube nuclear grade grease (nuclear grease), was introduced in this study.

## Results and Discussion

### Crystal structures for lysozyme and thaumatin

**Figure 1:**
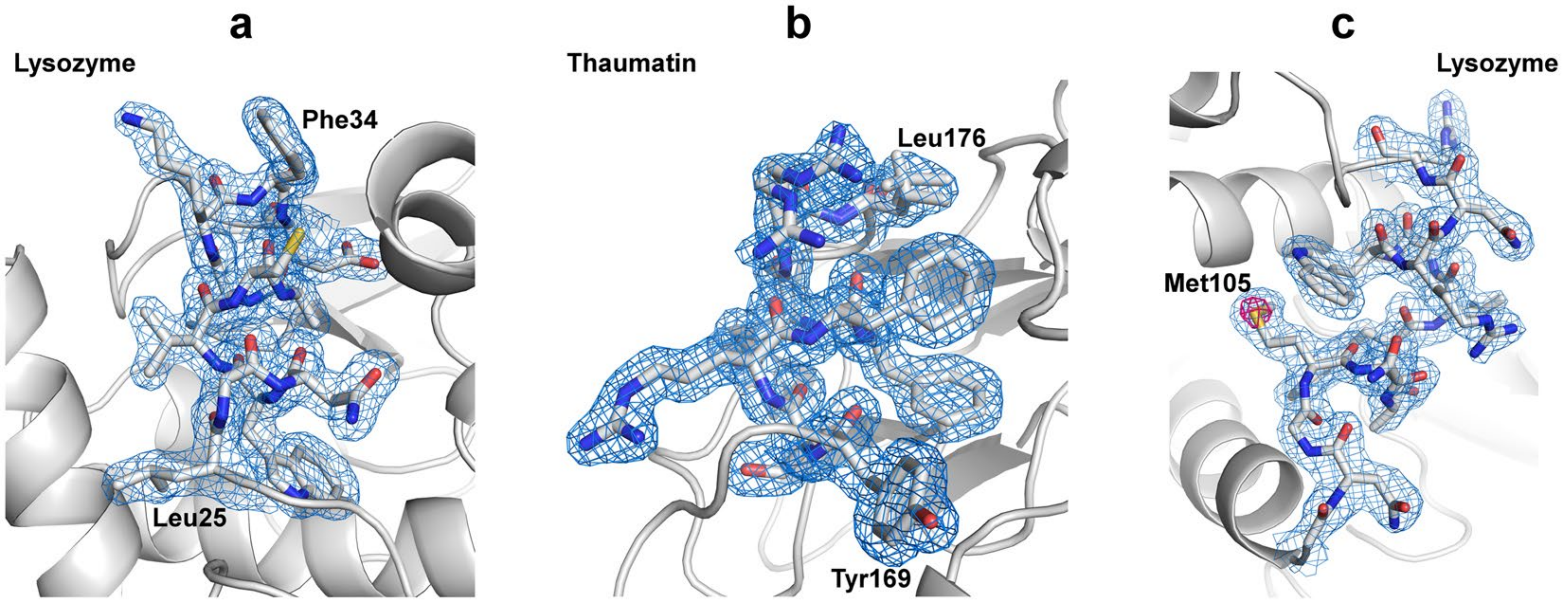
Electron density maps of lysozyme and thaumatin. Close-up views of (**a**) the lysozyme structure at 1.8-Å resolution and (**b**) the thaumatin structure at 1.55-Å resolution for the sample delivered in a cellulose matrix and (**c**) the lysozyme structure at 2.0-Å resolution for the sample delivered in a nuclear grease matrix with 2*F*_o_ – *F*_c_ electron density maps contoured at the 1.0 σ level (coloured blue). An anomalous difference Fourier map contoured at the 4.0 σ level (coloured magenta) shows the sulfur atom of Met105 in (**c**). These figures were drawn with PyMol (http://www.pymol.org).

We performed SFX experiments using femtosecond X-ray pulses from SACLA. Using lysozyme (1 × 1 × 1 μm) and thaumatin (2 × 2 × 4 μm) crystals (Supplementary Fig. 1a,b) dispersed in a cellulose matrix, we were able to collect 100,000–150,000 images in approximately 60–80 min at a wavelength of 1.24 Å (Table 1). At a flow rate of 0.43 and 0.47 μl min^−1^, a total sample volume of about 30–40 μl was used with a crystal number density of 5.8 × 10^8^ crystals ml^−1^ for lysozyme, and 4.3 × 10^8^ crystals ml^−1^ for thaumatin. We indexed and integrated 30,000–40,000 images for both the lysozyme (space group *P*4_3_2_1_2) and thaumatin (space group *P*4_1_2_1_2) crystals. The lysozyme and thaumatin crystals yielded data sets at 1.8-Å and 1.55-Å resolution with a completeness of 100% and a CC_1/2_ of 0.992 and 0.988, respectively. We determined and refined the crystal structures of lysozyme [Protein Data Bank (PDB) ID: 5wr9] and thaumatin (PDB ID: 5wr8) at 1.8-Å and 1.55-Å resolution (Fig. 1a,b), respectively. For the larger lysozyme crystals of the size 20 × 20 × 30 μm, the X-ray wavelength was kept at 0.95 Å. The microcrystals were used to acquire data sets at 1.45-Å resolution with a completeness of 100% and a CC_1/2_ of 0.995 (PDB ID: 5wra, Table 1).

**Table 1:** Crystallographic statistics. Values in parentheses are for the outermost shell.

Protein	Lysozyme			Thaumatin	
Carrier	16% cellulose	11% cellulose	Nuclear grease	22% cellulose	
Crystal density (crystals/ml)	5.8 × 10^8^	1.7 × 10^7^	2.4 × 10^8^	4.3 × 10^8^	
Crystal size (μm)	1 × 1 × 1	20 × 20 × 30	5 × 5 × 5	2 × 2 × 4	
Nozzle size (μm)	50	130	100	50	
Flow rate (μl/min)	0.43	0.75	0.42	0.47	
**Data collection**					
wavelength (Å)	1.24	0.95	1.77	1.24	
Space group	*P*4_3_2_1_2	*P*4_3_2_1_2	*P*4_3_2_1_2	*P*4_1_2_1_2	
**Unit-cell parameter**					
*a* = b (Å)	80.0	79.6	79.6	58.5	
*c* (Å)	38.4	38.3	38.2	151.6	
Number of collected images	149,938	107,856	105,769	101,383	
Number of hits	41,575	58,321	30,929	55,751	
Number of indexed images	29,593	40,787	19,271	43,350	
Indexing rate from hits (%)	71.2	69.9	62.3	77.8	
Number of merged images	29,593	40,787	19,271	43,350	
Number of total reflections	4,823,284	21,187,517	3,440,102	24,822,961	
Number of unique reflections	12,068	22,415	8,750	38,328	
Resolution range (Å)	30–1.8 (1.86–1.80)	30–1.45 (1.50–1.45)	30–2.0 (2.07–2.00)	30–1.55 (1.60–1.55)	
Completeness (%)	100 (100)	100 (100)	100 (100)	100 (100)	
Multiplicity	399.7 (283.0)	945.2 (677.3)	393.2 (81.9)	647.6 (668.5)	
*R*_split_ (%)^†^	7.1 (51.0)	5.1 (50.4)	8.0 (53.0)	8.6 (33.4)	
CC_1/2_	0.992 (0.764)	0.995 (0.735)	0.988 (0.654)	0.988 (0.760)	
<*I*/*σ*(*I*)>	10.2 (2.2)	13.4 (2.2)	10.5 (2.0)	7.7 (2.0)	
Total amounts of protein used (mg)	0.7	0.9	0.5	0.5	
**Refinement**					
*R*/*R*_free_ (%)	17.5/18.4	18.1/19.6	18.1/20.2	12.7/15.1	
R.m.s. deviations					
Bond lengths (Å)	0.008	0.007	0.008	0.006	
Bond angles (°)	1.059	1.071	1.070	0.984	
PDB code	5wr9	5wra	5wrb	5wr8	
**Protein**	**proteinase K**				
Carrier	16% cellulose (Pr)			16% cellulose (native)	
Crystal density (crystals/ml)		9.3 × 10^7^		4.9 × 10^7^	
Crystal size (μm)		4 × 4 × 4–5 × 5 × 7		8 × 8 × 8–12 × 12 × 12	
Nozzle size (μm)		50		110	
Flow rate (μl/min)		0.47		0.38	
**Data collection**					
wavelength (Å)		1.24		0.95	
Space group		*P*4_3_2_1_2		*P*4_3_2_1_2	
**Unit-cell parameter**					
*a* = b (Å)		68.6		68.3	
*c* (Å)		108.8		108.4	
Number of collected images		180,000		145,000	
Number of hits		40,503		59,246	
Number of indexed images		30,930		47,503	
Indexing rate from hits (%)		76.4		80.1	
Number of merged images	30,000	3,000	1,000	32,000	1,000
Number of total reflections	16,961,902	1,540,467	520,503	18,624,772	545,845
Number of unique reflections	42,391	42,386	42,060	42,385	42,273
Resolution range (Å)	32.7–1.50 (1.53–1.50)		27.2–1.50 (1.53–1.50)		
Completeness (%)	100 (100)	100 (99.9)	99.2 (93.3)	100 (100)	99.7 (99.6)
Multiplicity	400.1 (151.3)	36.3 (13.4)	12.4 (4.8)	439.4 (312.0)	12.9 (9.1)
*R*_split_ (%)^†^	7.8 (44.5)	24.4 (99.7)	43.1 (120.8)	7.1 (40.9)	41.4 (189.7)
CC_1/2_	0.990 (0.776)	0.896 (0.389)	0.713 (0.272)	0.992 (0.810)	0.761 (0.124)
<*I*/*σ*(*I*)>	10.2 (2.3)	3.7 (1.3)	2.5 (1.5)	10.9 (2.8)	2.3 (0.9)
Total amounts of protein used (mg)	0.9	0.09	0.03	0.4	0.01
**Refinement**					
*R*/*R*_free_ (%)	17.6/19.3				
**R.m.s. deviations**					
Bond lengths (Å)	0.009				
Bond angles (°)	1.004				
PDB code	5wrc				

^†^ $${R}_{s{\rm{plit}}}=1/\sqrt{2}\frac{{\sum }_{hkl}|{I}_{even}-{I}_{odd}|}{1/2\,{\sum }_{hkl}|{I}_{even}+{I}_{odd}|}$$.

**Figure 2:**
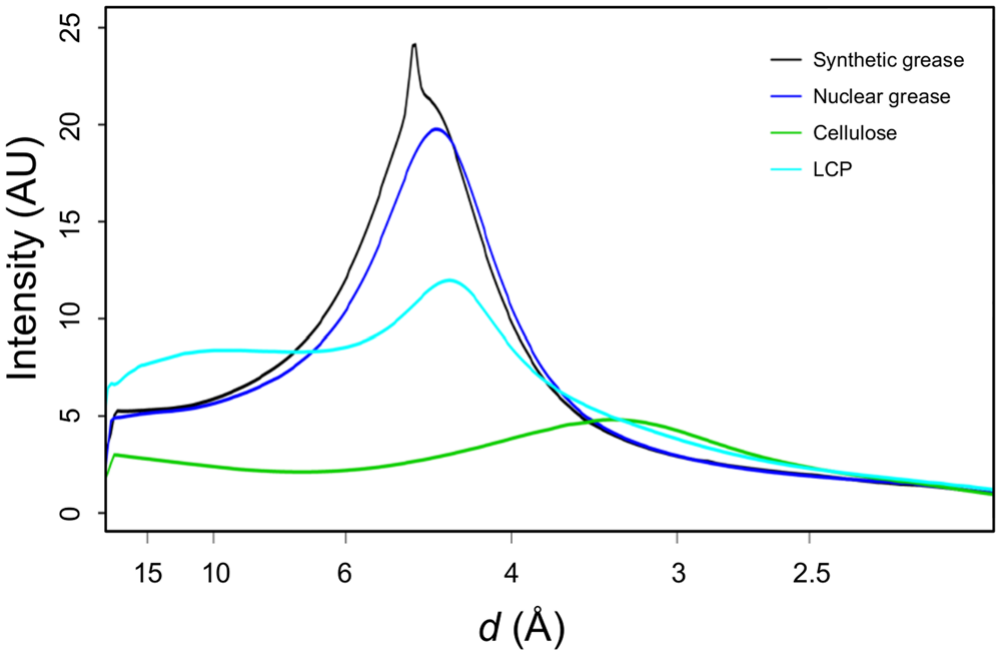
The average background scattering intensities of ~2,000 images from each matrix. Super Lube synthetic grease, Super Lube nuclear grease, 16% (*w/v*) hydroxyethyl cellulose solution and LCP are depicted by the black, blue, green and cyan lines, respectively.

In this study, 16% (*w/v*) and 22% (*w/v*) cellulose matrices were used for the small sized lysozyme (1 × 1 × 1 μm) and thaumatin (2 × 2 × 4 μm) crystals, respectively. The cellulose matrix with randomly oriented crystals was extruded through an injector nozzle with an inner diameter (i.d.) of 50 μm. On the other hand, for the larger lysozyme crystals (20 × 20 × 30 μm), an 11% (*w/v*) cellulose matrix was extruded through a 130-μm-i.d. nozzle. The cellulose matrix formed a stable flow for all protein samples (an example: Supplementary Fig. 2a). The matrix at low cellulose concentrations (less than ~5%) cannot be extruded from our injector system as a continuous sample column. On the other hand, a matrix at a cellulose concentration (~30%) becomes too hard for micro-extrusion. The cellulose concentration therefore was preferably ~10–20%. The sample preparation in our technique can be performed by simply mixing with matrix medium. Although the medium mixing technique using a syringe coupler may prevent crystal dehydration^23, 29^, the technique could cause mechanical damage to brittle crystals by physical contact between the crystals and the coupler interior surface, resulting in a deterioration of diffraction quality. In such cases, a simple, quick mixing with a spatula on a glass slide^21^ would be better to preserve the crystals. The cellulose matrix has lower background scattering (Supplementary Fig. 3a) compared to the conventional grease matrix, the synthetic grease Super Lube (Supplementary Fig. 3b) generated diffuse scatterings in the resolution range of 4–5 Å, and LCP^14^ (Fig. 2), while the cellulose matrix gives a slightly higher background scattering in the resolution range of ~3.5–2.5 Å. There were no significant differences between cellulose and hyaluronic acid matrices^26^, suggesting that polysaccharide hydrogels tend to have lower background scattering. However, the cellulose matrix is less adhesive than the hyaluronic acid matrix and prevents clogging of the sample-vacuum nozzle as a sample catcher^22^ (Supplementary Fig. 2) and adhesion of the matrix to the injector nozzle surface in our injector system. In addition, hyaluronic acid is more expensive compared to hydroxyethyl cellulose, up to ~1,000 times the price per gram. Hydrogels, LCP and Vaseline can be extruded as a continuous column with an approximately same diameter as a 50-μm-i.d. (or less) injector nozzle size. On the other hand, grease matrix tends to produce a column larger than the nozzle i.d. A sample column with a smaller diameter (~50 μm) contributes to the reduction of sample consumption and background scattering from the matrix^26^. A matrix with low background scattering is important to collect a high-resolution data set from ~1 μm (or less) crystals.

### *De novo* phasing

**Figure 3:**
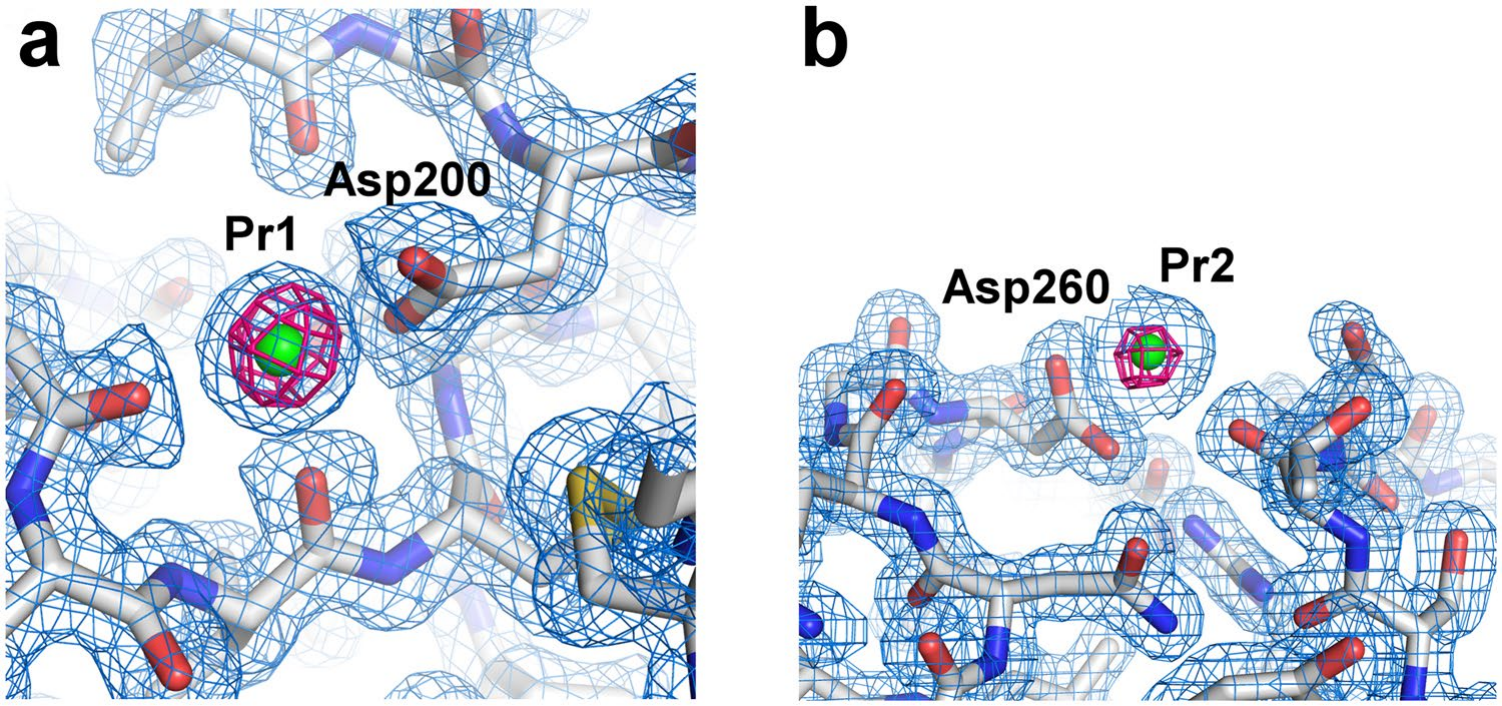
Electron density maps of proteinase K. (**a,b**) Close-up views of Pr ion binding sites with 2*F*_o_ – *F*_c_ electron density maps contoured at the 1.0 σ level (coloured blue). Bound Pr ions are depicted as a green sphere. The anomalous difference Fourier maps using 3,000 images (contoured at the 6.0σ level) are shown in magenta. These figures were drawn with PyMol (http://www.pymol.org).

Crystallographic phasing for routine structure determination remains a challenge in SFX. In this study, using the cellulose matrix, we attempted the *de novo* phasing of proteinase K. We collected ~180,000 images from the microcrystals (size 4 × 4 × 4–5 × 5 × 7 μm) of Pr-derivatized proteinase K (Supplementary Fig. 1c) at a wavelength of 1.24 Å (Table 1). We successfully indexed and integrated approximately 31,000 images in space group *P*4_3_2_1_2. The dataset extended to 1.5-Å resolution with a completeness of 100% and a CC_1/2_ of 0.990. The overall <*I*/σ(*I*)> of the merged observations was 10.2. Substructure determination and phasing were performed by *SHELXD* and *SHELXE* ^30^. We succeeded in locating two Pr ions in the asymmetric unit and could solve the substructure at 2.0-Å resolution, but not at 2.2-Å resolution. The two Pr-binding sites were identical to those of the calcium ions in the native structure (Fig. 3), indicating that the two calcium atoms were replaced by the Pr atoms^31^. The coordinates of the heavy atoms were employed for both the refinement and the phase calculation at 1.8-Å resolution in *SHEXLE*. A polyalanine model of proteinase K was automatically traced by *SHELXE*. Subsequently, 99% (277 of 279 residues) of the structure was automatically modelled with side chains by *Buccaneer* ^32^. Finally, we refined the structure at 1.5-Å resolution to an *R*/*R*_free_ of 17.6/19.3% (PDB ID: 5wrc). The expected magnitude of the anomalous signal (<|Δ*F*_ano_|>/<|*F*|>) is ~4.8% at 10 keV based on the formula in Hendrickson & Teeter^33^ and Dauter *et al*.^34^.

We found that 3,000 indexed images were sufficient for SAD phasing of proteinase K crystals. In this phasing, we used the first 3,000 of 30,930 indexed images, without deliberate selection of the best images. *SHELXD* located only one Pr atom in the asymmetric unit, when 3,000 indexed images were used. A polyalanine model from *SHELXE* at 1.7-Å resolution was completed in *Buccaneer*. We obtained 99% of the complete model. The final anomalous difference Fourier maps using 3,000 images in Fig. 3 display significant anomalous peak heights (17.1 and 11.2σ, obtained from *ANODE* ^35^) of the two Pr atoms.

**Figure 4:**
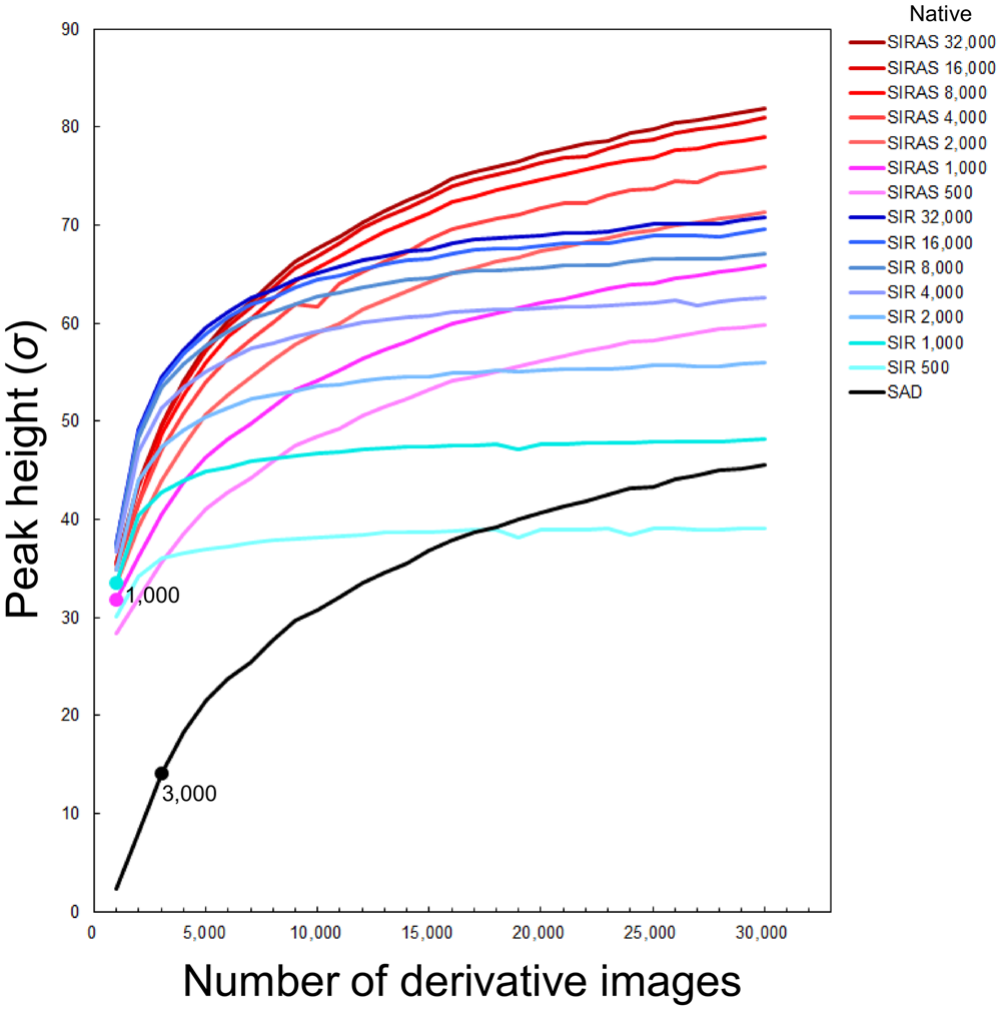
Improvement of anomalous difference peak heights with the number of derivative images. The plot of the sum of the anomalous peak heights from two Pr atoms. SAD, SIR and SIRAS phasing are shown in the black, blue and red lines, respectively. For SIR and SIRAS phasing, the number of native images were varied from 500 to 32,000. Filled circles indicate the minimum number of derivative images necessary for each phasing method and number of native images.

Next, we employed single-isomorphous replacement (SIR) and SIR with anomalous scattering (SIRAS) for phasing. We obtained a data set (32,000 indexed images) from native crystals of proteinase K at a wavelength 0.95 Å^36^, at a different beam time using different crystallization batches, at 1.5-Å resolution with a completeness of 100%, a CC_1/2_ of 0.992. Only 2,000 images in total (native/derivative: 1,000/1,000) were sufficient for SIR and SIRAS phasing of proteinase K, while SAD phasing required 3,000 images. The CC_1/2_ value of the 1,000-image derivative dataset was only 71.3% (27.2% for 1.53–1.50 Å), while that of the full dataset was 99.0% (77.6% for 1.53–1.50 Å) (Supplementary Fig. 4). As shown in Fig. 4, a combination of the native dataset with the derivative dataset boosted the peak heights in the anomalous difference map and allowed phasing from fewer images than using derivative images alone. This is in good agreement with the result from the previously reported I-SAD phasing of a membrane protein bacteriorhodopsin using an iododetergent^37^.

In SFX, *de novo* phasing for heavy atom-derivatized proteions has been demonstrated^16, 37–42^. In addition, native sulfur SAD phasing was also achieved^40, 43, 44^. These results indicate that *de novo* phasing is now routinely available for SFX. Our cellulose matrix with low background scattering noise is compatible with the accurate measurement of weak anomalous signals essential for *de novo* phasing from SFX data.

### A novel grease matrix with low background scattering

To reduce background scattering from conventional grease matrix^21, 26^, we introduced a novel grease matrix, Super Lube nuclear grade approved grease (nuclear grease). For lysozyme crystals (5 × 5 × 5 μm), we were able to collect ~100,000 images in approximately 1 hour at a wavelength 1.77 Å (Table 1). We indexed and integrated ~19,000 images for the lysozyme crystals. The crystals yielded data sets at 2.0-Å resolution with a completeness of 100% and a CC_1/2_ of 0.988. We determined and refined the crystal structure of lysozyme (PDB ID: 5wrb) at 2.0-Å resolution.

The conventional grease matrices (mineral-oil based AZ grease and untreated Super Lube synthetic grease without grinding treatment) extruded through a 110-μm-i.d. nozzle tended to produce a larger-diameter grease column (approximately ~210 μm) about the size of the outer diameter (o.d.) of the nozzle^21, 26^. On the other hand, the nuclear grease matrix was extruded as a continuous column with a diameter of ~100 μm through a 100-μm-i.d. nozzle (Supplementary Fig. 2b). The Super Lube synthetic grease tended to give a stronger diffraction ring at ~4.8-Å resolution in about 30% of all diffraction images (Fig. 2 and Supplementary Fig. 3b)^26^. Weaker background scattering was noted when using nuclear grease compared with Super Lube synthetic grease (Fig. 2 and Supplementary Fig. 3c). In the lysozyme structure with the nuclear grease matrix, we observed a weak anomalous scattering signal from sulfur atoms (e.g. the sulfur atom of Met105, Fig. 1c). On the other hand, an anomalous signal from the sulfur atoms in the proteinase K structure from ~20,000 indexed images was not discernible when using the conventional Super Lube synthetic grease matrix^26^. Using a wide variety of proteins, the adaptability of grease matrix has been demonstrated in SFX^15, 16, 18, 21, 26, 37, 39, 43, 45^. These results suggest that grease has potential as a versatile matrix carrier, but some crystals are incompatible with the grease matrix. The cellulose and hyaluronic acid matrices provide alternatives for grease-sensitive protein crystals. Grease and hydrogel crystal carriers are thus complementary (Table 2).

**Table 2:** Crystal carrier media for serial sample loading.

Media	Advantages	Disadvantages	References
Oil	general versatility	higher background scattering	
Nuclear grade grease	lower background scattering among grease matrices	salt-like impurities in grease	this study
Synthetic grease	higher versatility	gives a stronger diffraction ring at ~4.8 Å	26
Mineral-oil based grease	higher versatility	a larger diameter sample column	21
Vaseline (petroleum jelly)	a smaller diameter sample column (~40 μm)	gives stronger diffraction rings at 4.2 and 3.8 Å	22
Hydrogel	lower background scattering	damage to crystals by osmotic shock	
Hydroxyethyl cellulose	simple preparation	adhesive	this study
Hyaluronic acid	simple preparation	strongly adhesive, expensive	26
Agarose	compatible with proteins	requires heat treatment at temperatures higher than 85 °C as a pre-preparation	23
Other			
LCP (e.g., monoolein)	applicable to soluble and membrane proteins	higher background scattering, but lower than grease in the resolution range of 4–5 Å	20, 22, 23

Using the cellulose matrix as a general protein carrier, we obtained the structures of soluble proteins beyond 1.8-Å resolution at room temperature. We have successfully applied Pr-SAD, SIR and SIRAS phasing to SFX, using 3,000 indexed images for SAD and 2,000 images for SIR and SIRAS, demonstrating that we can accurately measure anomalous signals. Matrix carriers with a stable sample flow and a small diameter sample column have various application in SFX experiments such as femtosecond to millisecond time-resolved studies of light-driven structural changes, and chemical dynamics using pump-probe techniques^14, 18, 46–50^.

## Materials and Methods

### Sample preparation

Using a 20 mg ml^−1^ lysozyme solution, the crystals with a size of 1 × 1 × 1 μm, 5 × 5 × 5 μm and 20 × 20 × 30 μm were prepared following previously reported protocols^21^, except for the incubation temperature during crystallization at 12, 17 and 26 °C for 10 min, respectively. Thaumatin I was purified from crude thaumatin powder as described previously^51^. Thaumatin crystallization was performed using the batch method. Microcrystals (2 × 2 × 4 μm) were obtained by mixing in an ice bath an equal volume of the 40 mg ml^−1^ protein solutions and the reservoir solution, which consisted of 20 m*M* N-(2-acetamido) iminodiacetic acid (ADA) and 2.0 *M* potassium sodium tartrate (pH 7.3). Proteinase K from *Engyodontium album* (No. P2308, Sigma) at a concentration of 40 mg ml^−1^ was crystalized by previously reported protocols^26^. For Pr-derivatized proteinase K, a 100 μl sample of the crystal solution was added to a 100 μl heavy-atom solution comprised of 50 m*M* PrCl_3_, 0.5 *M* NaNO_3_ and 50 m*M* MES–NaOH (pH 6.5). The solution was then incubated at 20 °C for 90 min. To determine a crystal number density of the crystal solution, we counted the number of crystals in the solution using a hemocytometer (OneCell, cat. no. OC-C-S02) under a Hirox digital microscope (Hirox, KH-8700). The crystal number density was adjust to an approximately 10^7^–10^8^ crystals ml^−1^.

In this study, we used hydroxyethyl cellulose (mw ~250,000, No. 09368, Sigma) as the crystal carrier matrix. Protein microcrystals were prepared according to the following procedures. For lysozyme and proteinase K crystals, after a 100-μl sample of storage solution was centrifuged at ~1,300–3,000 × *g* for 10 sec using a compact tabletop centrifuge, a 40-μl aliquot of supernatant solution was dispensed into 50 μl of 32% (*w/v*) hydroxyethyl cellulose aqueous solution for lysozyme (1 × 1 × 1 μm) and proteinase K, or 22% (*w/v*) hydroxyethyl cellulose aqueous solution for lysozyme (20 × 20 × 30 μm) on a glass slide and then mixed with a spatula for ~15 sec. After a 50-μl aliquot of the remaining supernatant solution was removed, a 10-μl aliquot of the crystal solution was dispensed into 90 μl of the hydroxyethyl cellulose solution and then mixed for ~15 sec. For thaumatin crystals, after a 100-μl sample of storage solution was centrifuged at ~1,300–3,000 × *g* for 10 sec using a compact tabletop centrifuge, a 90-μl aliquot of supernatant solution was removed. A 10-μl aliquot of the crystal solution was dispensed into 90 μl of 24% (*w/v*) hydroxyethyl cellulose aqueous solution on a glass slide and then mixed for ~15 sec. For the grease matrix, the lysozyme crystals (5 × 5 × 5 μm) were mixed with the Super Lube nuclear grade grease (No. 42150, Synco Chemical Co.) using the same procedure reported by Sugahara *et al*.^21^ The grease was filtered through 10 μm mesh (No. 06-04-0041-2314, CellTrics) before mixing with protein crystals to remove salt-like impurities in the grease. We performed this matrix preparation immediately before SFX experiments.

### Data collection

We carried out the experiments using femtosecond X-ray pulses from SACLA^27^. The X-ray wavelength was 0.95, 1.24 or 1.77 Å (13, 10 or 7 keV) with a pulse energy of ~200 μJ. Each X-ray pulse delivers ~7 × 10^10^ photons within a 10-fs duration (FWHM) at a wavelength of 1.77 Å (7 keV) to the matrices. Data were collected using focused X-ray beams of 1.5 × 1.5 μm^2^ by Kirkpatrick-Baez mirrors^52^. The crystals in a cellulose or grease matrix were serially loaded using a high viscosity micro-extrusion injector system installed in a helium ambiance, diffraction chamber. The experiments were carried out using a Diverse Application Platform for Hard X-ray Diffraction in SACLA (DAPHNIS)^28^ at BL3^53^. The microcrystals embedded in the matrix were kept at a temperature of approximately 20 °C in the micro-extrusion injector. The sample chamber was kept at a temperature of ~26 °C and a humidity greater than 50%. Diffraction images were collected using a custom-built 4M pixel detector with multi-port CCD sensors^54^. The matrix with randomly oriented crystals was extruded through injector nozzles with inner diameters (i.d.) of 50, 100, 110 or 130 μm (Table 1). Data collection was guided by realtime analysis by the SACLA data processing pipeline^55^.

### Background intensity determination

The background intensities from Super Lube synthetic grease, Super Lube nuclear grease and hydroxyethyl cellulose through a 100-μm-i.d. nozzle at 1.77 Å and that from LCP^14^ through a 75-μm-i.d. nozzle at 1.61 Å were determined by a procedure similar to that used in Conrad *et al*.^23^ Details of the calculation have been described previously^26^. Diffraction images for LCP were retrieved from CXIDB^56^ (http://www.cxidb.org/) #53.

### Structure determination

Diffraction images were filtered and converted by *Cheetah* ^57^ adapted^55^ for the SACLA data acquisition system^58^. Diffraction peak positions were determined using the built-in Zaefferer algorithm and passed on to *DirAx* ^59^ for indexing. No sigma cutoff or saturation cutoff were applied. Measured diffraction intensities were merged by *process_hkl* in the *CrystFEL* suite^60^ with scaling (–*scale* option). The structures of lysozyme and thaumatin were determined by difference Fourier synthesis using search models (PDB: 3WUL for lysozyme, and 3X3P for thaumatin). For Pr-derivatized proteinase K, substructure search, phasing and phase improvement were carried out using the *SHELX C, D* and *E* programs^30^. The autotraced model from *SHELXE* was fed into *Buccaneer* ^32^ from the *CCP4* suite^61^. Manual model revision and structure refinement were performed using *Coot* ^62^ and *PHENIX* ^63^, respectively. Details of the data collection and refinement statistics are summarized in Table 1.

## Electronic supplementary material

Supplementary Figures
