# Supplementary material for: Hydroxyethyl cellulose matrix applied to serial crystallography
Source: Sci Rep. 2017 Apr 6;7:703. doi: 10.1038/s41598-017-00761-0 (PMC5429652; doi:10.1038/s41598-017-00761-0)
Supplement: Supplementary file 1 — Supplementary Figures [file 41598_2017_761_MOESM1_ESM.pdf]

Hydroxyethyl cellulose matrix applied to serial crystallography

Michihiro Sugahara*, Takanori Nakane, Tetsuya Masuda, Mamoru Suzuki, Shigeyuki Inoue, Changyong Song, Rie Tanaka, Toru Nakatsu, Eiichi Mizohata, Fumiaki Yumoto, Kensuke Tono, Yasumasa Joti, Takashi Kameshima, Takaki Hatsui, Makina Yabashi, Osamu Nureki, Keiji Numata, Eriko Nango, So Iwata

Supplementary Figures

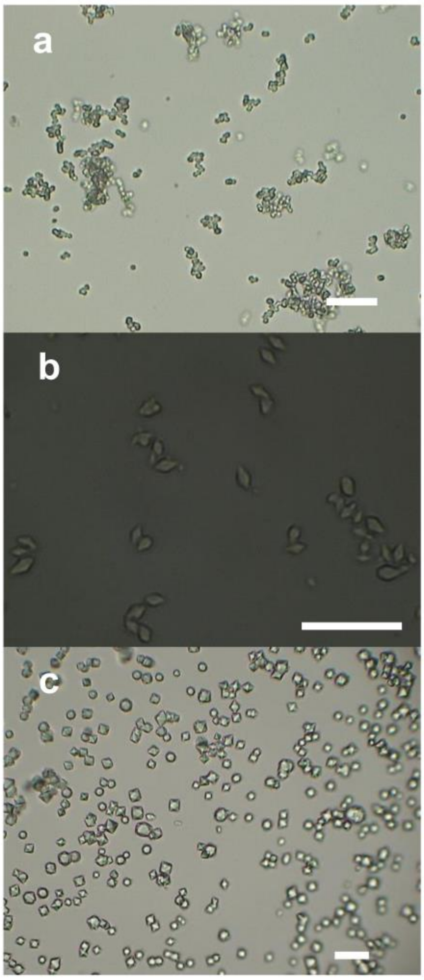

Supplementary Figure 1: Protein microcrystals used for SFX measurements. (**a**) lysozyme, (**b**) thaumatin and (**c**) proteinase K crystals. Scale bars represent 20 µm.

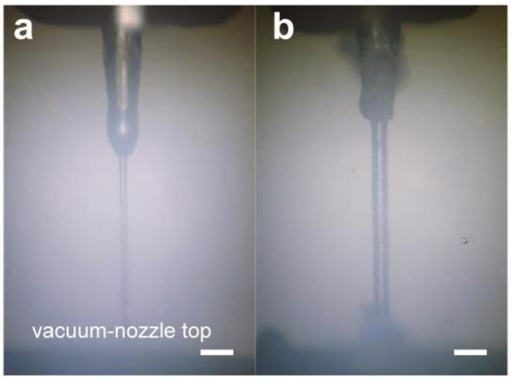

Supplementary Figure 2: Sample extrusion of the two crystal carriers. (**a**) hydroxyethyl cellulose and (**b**) Super Lube Nuclear grease were extruded as a continuous column to intersect with the XFEL beam through a 50- and 100-µm-i.d. nozzle, respectively. Scale bars represent 200 µm.

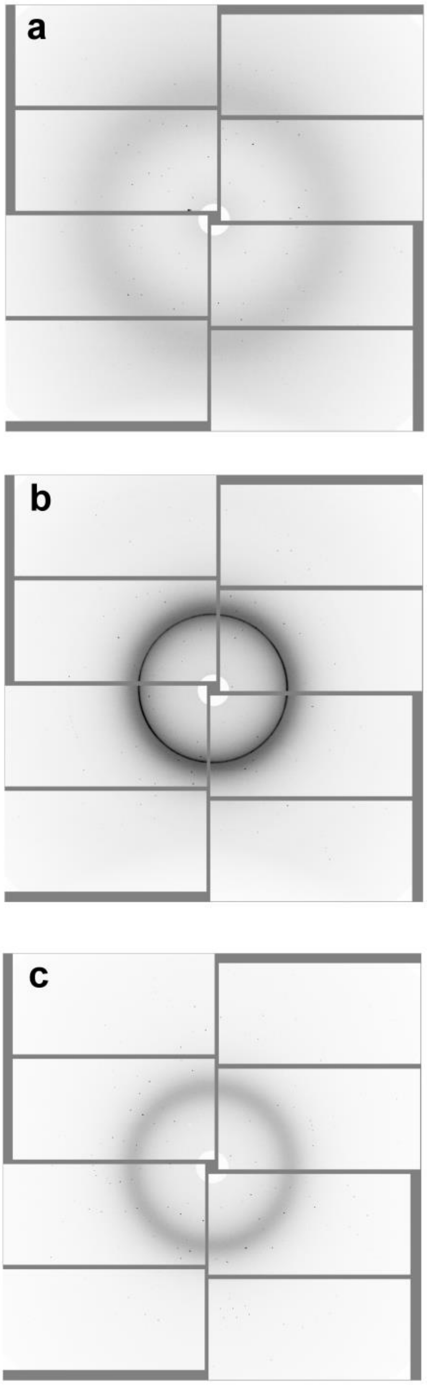

Supplementary Figure 3: Typical XFEL single diffraction. (**a**) cellulose matrix, (**b**) Super Lube synthetic grease and (**c**) Super Lube nuclear grease.

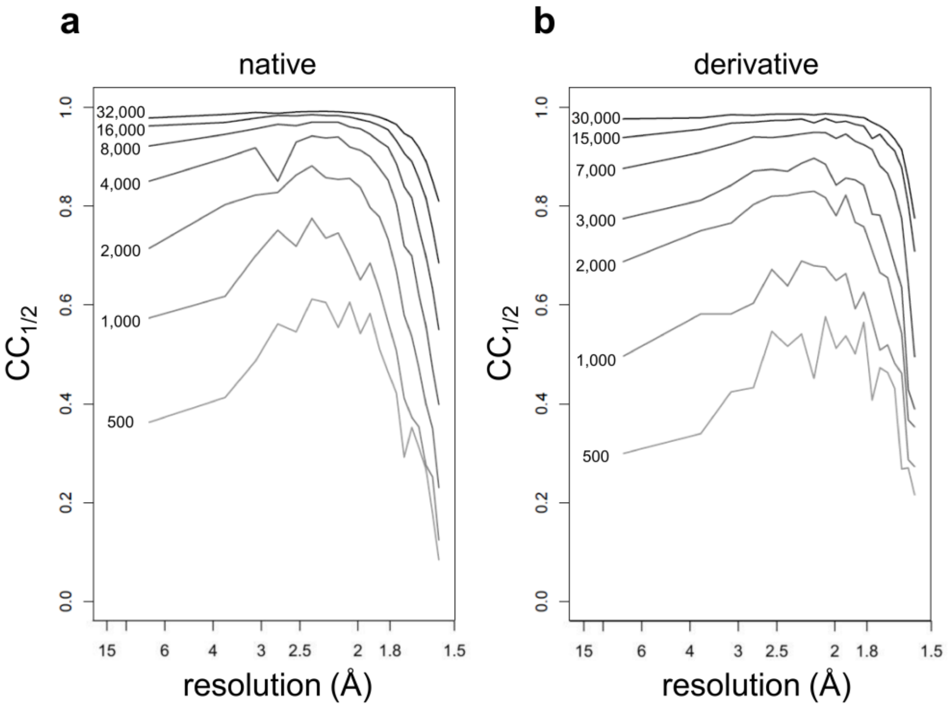

Supplementary Figure 4: $CC_{1/2}$ of the proteinase K datasets for different numbers of indexed images. (**a**) native and (**b**) Pr-derivative.
